# Chronotype and depression in adolescence: Results from a UK birth cohort study

**DOI:** 10.1002/jcv2.12245

**Published:** 2024-05-10

**Authors:** Dimitris I. Tsomokos, Elizabeth Halstead, Eirini Flouri

**Affiliations:** ^1^ School of Psychology and Neuroscience University of Glasgow Glasgow UK; ^2^ Sleep Education and Research Laboratory UCL Institute of Education University College London London UK; ^3^ Psychology and Human Development UCL Institute of Education University College London London UK

**Keywords:** adolescent mental health, chronotype, circadian rhythms, depression, eveningness, sex differences, sleep

## Abstract

**Background:**

Research has established a bidirectional association between sleep disturbances and depression in both adults and youth, as well as links between depression and circadian rhythms and chronotype, predominantly in adult populations. However, the link between chronotype and depression in the general adolescent population, independently of poor sleep and prior mental health problems, remains unclear.

**Methods:**

This study investigated the association between time‐to‐sleep (TTS) and depressive symptoms in middle adolescence (age 14 years) using data from a large, nationally representative birth cohort from the UK. The relationship between TTS and self‐reported number of depressive symptoms was adjusted for individual, family, and neighborhood characteristics, including sleep quality, earlier mental health, diet and family meal routines, body‐mass index, screen time, physical activity, chronic illness, special educational needs, peer victimization, socioeconomic status, maternal mental health, area safety and the built environment (air pollution).

**Results:**

An “evening” chronotype was positively associated with depressive symptoms, and biological sex moderated this association—with eveningness being more strongly related to depressive symptoms in females. TTS inconsistency between non‐school and school nights was associated with depressive symptoms and sleeping later on non‐school nights predicted fewer depressive symptoms. The results were robust to further sensitivity analyses that used the sleep midpoint on non‐school nights and controlled for sleep duration.

**Limitations:**

This was a correlational study. The independent and dependent variables were self‐reported, and there was no clinical screening for sleep disorders. The TTS variables were provided in crude hour slots.

**Conclusions:**

A robust association was found between evening chronotype and depressive symptoms in middle adolescence, even after adjustment for a wide range of confounders. Eveningess and depressive symptoms were more strongly associated in females.

## Graphical abstract

Adolescents with an “evening” chronotype reported a higher number of depressive symptoms at age 14 years. Eveningness was more strongly related to depressive symptoms in females. The findings were robust even after adjustment for a wide range of environmental and biopsychosocial factors, both proximal and distal, including poor sleep quality and prior mental health problems.

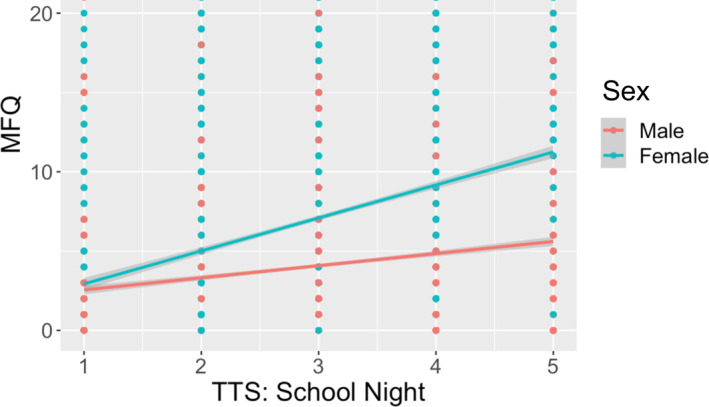

**Key points**

- In recent years, much has been known about the links between chronotype and depression in adults, but not in the general adolescent population independently of poor sleep, sleep duration, and prior mental health.
- We investigated the relationship between a proxy measure of chronotype (time‐to‐sleep) and self‐reported number of depressive symptoms at age 14 years, in a large UK birth cohort, which allowed us to adjust for individual, family, and neighborhood confounders.
- A robust association was found between evening chronotype and depressive symptoms in middle adolescence, even after adjustment for a wide range of confounders.
- Eveningness and depressive symptoms were more strongly associated in females.

## INTRODUCTION

Clinically significant symptoms of depression affect 1 in four youth around the world (Racine et al., 2021), with even subclinical depressive symptoms now being characterized as a major public health concern (Auerbach et al., 2018; Bitsko et al., 2022; Newlove‐Delgado et al., 2021; Ravens‐Sieberer et al., 2022). In the United States, for example, the Centers for Disease Control and Prevention (CDC) have estimated that 42% of adolescents aged between 14 and 18 years experienced depressive symptoms in 2021, up from 37% in 2019 (CDC, 2023; Jones et al., 2022). Even before the COVID‐19 pandemic, the prevalence of past‐year major depressive episodes for this age group in the US had risen to 16% overall, and 23% for females (Daly, 2022). Adolescent depression has been associated with numerous negative outcomes later in life, is a leading source of disability worldwide, and is associated with diminished quality of life and morbidity (Clayborne et al., 2019).

The association between sleep patterns and adolescent depression has now been well established (Orchard et al., 2020; Riemann et al., 2020). It is also known that the sleep‐depression association may differ substantially by biological sex (Fabio Fabbian et al., 2016; Kivelä et al., 2018; Taylor & Hasler, 2018), with females at higher risk of both depression (Bone et al., 2020) and sleep disturbances (Markovic et al., 2020) and with greater vulnerability to mood deficits following sleep loss (Short & Louca, 2015). Recent evidence however has also highlighted the important role of chronotype in the association between sleep and depression, especially in adolescence (Bartel et al., 2015; Gradisar et al., 2022; Zou et al., 2022), when chronotype (sleep‐wake cycle) patterns shift towards eveningness (Gradisar et al., 2011). Chronotype captures an individual's endogenous preferred timing of sleep and wakefulness, and it is linked to entrainment—the process by which biological rhythms synchronize with external cues, such as light and darkness (Golombek & Rosenstein, 2010). Disruptions in circadian alignment, for example, by irregular sleep patterns or exposure to artificial light from screens, have been associated with physical but also mental health problems (Meyer et al., 2022; Potter et al., 2016). For example, a later sleep‐wake cycle, typically referred to as “evening” chronotype, has been associated with a range of risky behaviors and psychological problems (Hasler et al., 2013), including depressive symptoms, partly due to delayed sleep‐phase onset (Crowley et al., 2007; Sivertsen et al., 2013) and suboptimal sleep durations (Short et al., 2020). In adolescence, in particular, significant under‐sleeping compared to the average recommended range of 8–10 h for non‐clinical groups (Fuligni et al., 2019; Paruthi et al., 2016) has been shown to exert a causal influence on known risk factors for depression (Gradisar et al., 2022), such as mood valence (Baum et al., 2014; Talbot et al., 2010).

Despite this progress in research, there remains a distinct lack of large‐scale studies on the role of chronotype independently of sleep quantity or quality in the general youth population, especially during the critical transition period to middle adolescence (Haraden et al., 2017; Kivelä et al., 2018), when sex differences in depression emerge and both sexes shift to eveningness. The small‐ and medium‐scale studies that have addressed the issue have offered conflicting results: in a recent study of 329 adolescents, Karan et al. (2021) found that eveningness predicted more risk‐taking in males but was not associated with depressive symptoms in either sex. This was broadly in line with an earlier study (*N* = 47 adolescents) that had reported decreased positive affect—but not increased negative affect—in both sleep‐deprived and evening‐chronotype adolescents (Dagys et al., 2012). In other studies, however, recently reviewed by Gradisar et al. (2022), eveningness was found to precede the reduction of emotion regulation abilities during adolescence, leading to anxiety and depression via negative cognitions, rumination, and catastrophizing (Blake et al., 2018).

In the present work we aim to fill this gap by investigating the relationship between chronotype and depressive symptoms in middle adolescence using data from the UK's Millennium Cohort Study (MCS), a large, population‐based longitudinal birth cohort. We ask whether a proxy measure of chronotype, namely, time‐to‐sleep (TTS), is associated with the total depressive symptoms score from the Mood and Feelings Questionnaire (MFQ), both of which were self‐reported by cohort members at age 14 years, during the sixth sweep of the survey.

Given that in adolescence sleep and depressive symptoms have been associated not only with sex, as discussed, but also socioeconomic status and ethnicity (Marco et al., 2011; Patalay & Fitzsimons, 2018; Pine et al., 2022), screen time (Boers et al., 2019; Hisler et al., 2020; Kandola et al., 2022), physical activity (Gu, 2022; Slykerman et al., 2020), diet and family meal routines (Hoare et al., 2020; Moitra et al., 2020), body‐mass index (BMI) (Patalay & Hardman, 2019), alcohol consumption (Pasch et al., 2010), decision‐making and risk‐taking (Bartel et al., 2015; Bentivegna et al., 2022; Berdynaj et al., 2016), chronic illness and special educational needs (SEN) (Hysing et al., 2008; Pezzimenti et al., 2019), maternal mental health (Fitzsimons et al., 2017), peer victimization (Sharpe et al., 2022), seasonal characteristics (Kivelä et al., 2018; Wescott et al., 2022), and the built environment (Mayne et al., 2021; Mueller & Flouri, 2021), our analysis fully accounted for all these individual, family and contextual factors. Crucially, we also controlled for prior mental health difficulties (i.e., at the previous sweep at age 11 years) as well as sleep quality (sleep latency and number of night awakenings during the night).

We expected that eveningness would be related to depressive symptoms, but also that sex would moderate this association, such that the link would be stronger for females, who in general need more sleep and tend to have an earlier chronotype (F. Fabbian et al., 2016; Fischer et al., 2017; Randler & Engelke, 2019). Finally, to test the robustness of our results we performed two sensitivity analyses. First, to ensure that eveningness was not confounded by sleep quantity, we excluded adolescents who were under‐sleeping (or over‐sleeping), namely, those who slept less than 7 (or more than 11) hours. Second, instead of TTS we used a sleep midpoint measure on non‐school nights, in view of the evidence that this may be a better behavioral proxy of adolescent chronotype (Gauthier‐Gagné et al., 2023; Roenneberg et al., 2019; Terman et al., 2001).

## METHODS

### Analytic sample

The MCS tracks over 19,000 children in the UK born during 2000–2002 (Joshi & Fitzsimons, 2016), from around 9 months to around 3, 5, 7, 11, 14 and 17 years. Electoral wards provided the MCS sampling frame, over‐representing the smaller UK countries, as well as families living in wards of high child poverty across the UK, and wards with high proportions of ethnic minorities in England (Plewis et al., 2004). Data were collected via interviews with, and self‐completion questionnaires from, the main respondent (overwhelmingly the mother) in the child's home, as well as the cohort members themselves and their teachers, but also via other methods including tests and third‐party observations. Ethical approval was gained from UK Multi‐Centre Ethics Committees, and parents gave informed consent before interviews took place, as did the cohort children themselves from age 14 onwards. At the age 14 sweep of the survey, 11,726 families took part (11,872 adolescents). Data for this sweep were collected between January, 2015 and March, 2016. The present study's analytic sample included singletons and first‐born twins or triplets who had complete data on the independent variable: TTS during school nights and non‐school nights, provided in 1‐h slots (e.g., 9–9.59 p.m.). As a result, the final analytic sample in our study included 11,314 adolescents (50.2% female, 79% white; further details provided in Table 1).

**TABLE 1:** Sample bias: differences between the analytic sample and the rest of the MCS at age 14 on some key study variables (for all study variables see SOM).

	Analytic sample, *N* = 11,314 (97%)	Rest of MCS at age 14, *N* = 403 (3%)	Statistic	*p*
Categorical variables, *n* (%)				
Sex				
Female	5680 (50%)	158 (39%)	18.39	<0.001
Male	5634 (50%)	245 (61%)		
SEN				
Yes	465 (4%)	63 (16%)	117.41	<0.001
Chronic illness				
Yes	1655 (15%)	104 (26%)	58.13	<0.001
Maternal mental illness				
No	6579 (58%)	155 (39%)	9.65	0.002
Stratum				
England—Adv.	3143 (28%)	94 (23%)	12.21	0.142
England—Disadv.	2756 (24%)	118 (30%)		
England—Ethnic	1505 (13%)	56 (14%)		
Wales—Adv.	527 (5%)	15 (4%)		
Wales—Disadv.	1084 (10%)	42 (10%)		
Scotland—Adv.	670 (6%)	18 (4%)		
Scotland—Disadv.	548 (5%)	26 (6%)		
Northern Ireland—Adv.	441 (4%)	16 (4%)		
Northern Ireland—Disadv.	640 (6%)	18 (4%)		
Ethnicity				
White	8933 (79%)	31 (8%)	14.37	0.013
Mixed	530 (5%)	4 (1%)		
Indian	303 (3%)	0 (0%)		
Pakistani and Bangladeshi	817 (7%)	9 (2%)		
Black/Black British	361 (3%)	2 (0%)		
Other ethnic group	277 (2%)	0 (0%)		
(*Missing*)	93 (1%)	457 (89%)		
Numerical variables, mean (se)				
TTS: School night (1–5)	2.94 (0.01)	2.80 (0.53)	0.27	0.796
TTS: Non‐school night (1–5)	3.96 (0.01)	3.62 (0.29)	1.19	0.257
Income quintile (1–5)	3.19 (0.01)	2.59 (0.07)	8.42	<0.001
Maternal education (1–6)	3.90 (0.01)	3.30 (0.08)	6.95	<0.001
Prior mental health problems (SDQ, age 11) (0–36)	7.52 (0.06)	11.10 (0.42)	−8.37	<0.001

*Note*: Statistic and *p*‐values reported are for *χ* ^2^‐tests (*t*‐tests) for categorical (continuous) variables.

Abbreviations: Adv., Advantaged; Disadv., Disadvantaged; MCS, Millennium Cohort Study; SOM, supplemental online material.

### Measures

#### Depressive symptoms: MFQ

The outcome (dependent) variable was the adolescent's total score on the short form MFQ (Angold et al., 1995), a screening instrument composed of 13 self‐report items (e.g., “*I felt miserable or unhappy*” and “*I thought I could never be as good as other kids*”) scored from 0 to 2. A score of 0 corresponded to “Not true,” 1 to “Sometimes,” and 2 to “True.” Therefore, the total score ranged from 0 to 26, and higher scores corresponded to more depressive symptoms. A widely accepted cut‐off for the likely presence of clinical depression is a total score of 12 (Jarbin et al., 2020; Thabrew et al., 2018). Reliability of the MFQ scale in our sample was excellent, with Cronbach's *α* = 0.93.

#### Chronotype: TTS on school and non‐school nights

The independent variable, TTS, was the self‐reported time (in hour slots or ranges) of falling asleep. At age 14, cohort members were asked, “About what time do you usually go to sleep on a school night?” (“TTS: school night” variable). There were five possible answers: (1) before 9 p.m, (2) 9–9:59 p.m, (3) 10–10:59 p.m, (4) 11–midnight, or (5) after midnight. The same options were given for the question: “About what time do you usually go to sleep on the nights when you do not have school the next day?” (“TTS: non‐school night” variable). Therefore, both of these TTS variables were interval variables, with values from 1 to 5. The TTS variable for school nights (non‐school nights) has been shown to be strongly (moderately) correlated both with actigraphy and with well‐established sleep questionnaires at this age (Paciello et al., 2022).

#### TTS difference between non‐school nights and school nights

A third independent variable was derived from the difference between the TTS reported for non‐school nights and school nights, thus tracking the inconsistency between them (“TTS: inconsistency”). This was an interval variable with values from −4 to 4. A value of −4 implies that the non‐school‐night (Friday and Saturday) TTS was before 9 p.m whereas the school‐night TTS was after midnight; a value of 4 implies the opposite scenario. Therefore, as an example, if a cohort member goes to sleep at 9 p.m on a school night and 12 p.m on Friday or Saturday (non‐school night) then the TTS: inconsistency score is 3.

#### Midpoint of sleep on non‐school nights

As mentioned, in an additional (sensitivity) analysis we used sleep midpoint on non‐school nights as the main independent variable. This is considered by some researchers as a better proxy of chronotype in adolescence (Gauthier‐Gagné et al., 2023; Roenneberg et al., 2019; Terman et al., 2001). In this case, as wake‐up times were also given in “hour slots” in MCS, both TTS and wake‐up time were anchored at the middle of the range (e.g., 7:30 a.m for the 7–8 a.m hour slot), and the midpoint was calculated on that basis.

#### Confounders

As discussed, we adjusted for area, family, and individual covariates associated with both the independent and the dependent variable. The area‐level covariates were aspects of the built environment (air pollution) and the social environment, and perceived safety. Air pollution (ranging from 1 to 10, lowest to highest level), measured at ward‐level and converted to deciles, was the annual mean concentration of nitrogen dioxide (NO_2_) (Church & Midouhas, 2016). The social environment was approximated by the MCS sampling stratum (type of ward within a UK country) at the beginning of the survey. For each UK country, there was an advantaged and a disadvantaged stratum; in England, there also was an ethnic minority stratum. The “*Ethnic minority*” stratum comprised English wards that had an ethnic minority indicator of at least 30% in the 1991 Census, that is, at least 30% of their total population fell into the two categories “Black” (Black Caribbean, Black African and Black Other) or “Asian” (Indian, Pakistani and Bangladeshi). The “*Disadvantaged*” stratum included wards that were not part of the Ethnic minority stratum, and which fell into the upper quartile (poorest 25% of wards) of the ward‐based Child Poverty Index (Plewis et al., 2004). Area safety was reported by the parent in response to how safe they thought it was for their adolescent to walk or socialize within a mile radius or a 20‐min walk from home, with values from 1 (not at all safe) to 4 (very safe).

In terms of family characteristics at the age 14 sweep, we adjusted for *income* (given in OECD equivalised household income quintiles); *maternal education* (the highest educational level of the main respondent—the mother, in the vast majority—based on the UK's National Vocational Qualification and its equivalents and given as an interval variable from 1 to 6); *maternal mental illness* (whether or not the mother had ever been diagnosed with depression or anxiety); *family meals* (the frequency of the family having a meal together during the previous week, ranging from 1 to 4, i.e. from not at all to every day); and whether or not the cohort member had *moved school* (i.e., whether current school is different from the one they started at).

In terms of individual‐level characteristics we adjusted for biological *sex* (male or female) and *ethnicity* (as reported using the UK Census's 6‐group classification: White, Mixed, Indian, Pakistani and Bangladeshi, Black or Black British, or Other Ethnic group); whether or not the cohort member slept in a *shared bedroom*; whether or not they had a *chronic illness*; whether or not they had a statement of SEN and whether or not they consumed at least two portions of *fruit* and *vegetables* per day. We also adjusted for cognitive ability which at the age 14 sweep was vocabulary (*word score*, an interval variable ranging from 1 to 20), measured by showing a word (such as “conceal”) on a computer screen and asking the cohort member to pick the right synonym (“hide”) among several options (Sullivan et al., 2021)—with higher scores indicating a greater number of correct answers. We also controlled for *quality of decision‐making* at age 14, a continuous variable from 1 to 74 in our sample, that was drawn from the adolescent's score on the Cambridge Gambling Task, which assesses reward and punishment sensitivity under conditions of risk, or reward processing more broadly (CANTAB, 2006; Robbins et al., 1994). Here, again, higher scores correspond to better decision‐making under risk and uncertainty. Furthermore, we adjusted for the adolescent's self‐reported attitude to *risk‐taking* (“*how willing to take risks would you say you are*?,” interval variable from 1 to 11, never to always); *BMI* (continuous variable, objectively measured); self‐reported weekly frequency of moderate to vigorous *physical activity* (from 1, not at all, to 5, every day); self‐reported daily amount of *screen time* (from 1, none at all, to 8, which corresponded to 7 h or more); self‐reported *alcohol consumption* over the past 4 weeks (from 1, never, to 7, 20 times or more); and self‐reported frequency of being hurt or picked on by other children on a weekly basis (*bullied* variable, with values ranging from 1, never, to 6, most days). Furthermore, we adjusted for the *season* when the interview took place, as well as sleep quality using two variables: a self‐reported estimate of *sleep latency* (‘*During the last four weeks*, *how long did it usually take for you to fall asleep?*’, an interval variable from 1 to 5, corresponding to 0–15 min, 16–30 min, 31–45 min, 46–60 min, or over 60 min to fall asleep); and the number of *night awakenings* (‘*During the last four weeks*, *how often did you awaken during your sleep time and have trouble falling back to sleep again?*’, 1–6, corresponding to none, a little, some, a good bit, most, and all of the time). Finally, we adjusted for the cohort member's *prior mental health difficulties* at age 11 years using the parent‐reported Strengths and Difficulties Questionnaire (SDQ), as the MFQ was not available at the age 11 sweep of the survey. We used the SDQ's total difficulties scale (with scores ranging from 0 to 36, in our sample), which consisted of 20 items from four subscales, namely, peer and emotional (internalizing) problems, and conduct and hyperactivity‐inattention (externalizing) problems (Goodman, 1997). The SDQ's total difficulties score indexes the level of general socio‐emotional and behavioral problems (O’Connor et al., 2018; Stringaris et al., 2014), and its use here—i.e., without selecting SDQ scales for specific mental health problems—is justified in view of the substantial comorbidity within and heterotypic continuity of mental health problems in adolescence (Goodman et al., 2010; Ingoldsby et al., 2006; Kosterman et al., 2010; McDonough‐Caplan et al., 2018; Park & Chang, 2022).

### Analytic strategy

Analysis was carried out in four stages. Firstly, we examined the impact of sample selection and missing data. Secondly, we examined the bivariate associations between the main variables. Thirdly, we proceeded with survey‐weighted multiple regression models; missing data in these models were handled by multiple imputation (see next section). In the fourth and final stage, we performed an additional sensitivity analysis excluding cohort members who under‐slept or over‐slept, to ensure that any relationship between chronotype and depressive symptoms was not due to sleep duration. For non‐school nights, we also calculated a sleep midpoint (to the extent that this was possible given our data) and entered this variable alongside sleep duration in our models instead of TTS. Full details for the variables used, the analytic sample, and all stages of the analysis can be found in a Supplemental Online Material document (SOM, 2023).

#### Sample bias and missing data

We performed descriptive analyses, firstly to identify any differences between participants in the analytic sample and those excluded from it and, secondly, to ensure that missingness in our analytic sample was both low and random. Our test examining whether data were missing at random (MAR) or if there was another pattern of missingness also informed the subsequent imputation process. Descriptive analyses were performed without using survey weights (“unweighted” analyses), as the purpose was to describe missingness and any systematic bias in the analytic sample, not make statistical inferences (by contrast, in the case of multiple regression models below, we applied survey weights).

#### Bivariate and correlation analyses

To gain a better understanding of the raw data, we produced scatterplots of MFQ scores against time‐to‐sleep (TTS: school night), stratifying by sex. Simple bivariate analyses followed, and a correlation table was produced for the main numerical variables used in the study.

#### Regression models

All models were survey‐weighted and fitted on multiply imputed data. We started with an unadjusted linear regression model, which included only area stratum as a covariate, then entered additional covariates progressively in three different models. Our final, fully adjusted model was fitted both with and without the interaction between sex and chronotype. Therefore, the baseline model Equation (1) was given by:

(1) $$\text{MFQ}=a+b_{1}\cdot \text{TTS}+b_{2}\cdot \text{Stratum}$$

(Note that we start from a model in which the only covariate is area stratum as MCS is a stratified sample.) In the second model Equation (2), we added sex, ethnicity, income, and maternal education:

(2) $$\text{MFQ}=\left(1\right)+b_{3}\cdot \text{Sex}+b_{4}\cdot \text{Ethnicity}+b_{5}\cdot \text{Income}+b_{6}\cdot \text{Mat}.\;\text{Edu}.$$

In the third step, model (3), we added word score, quality of decision‐making, risk‐taking, presence of chronic illness, BMI, fruit and vegetable consumption, physical activity, screen time, alcohol consumption, season, peer victimization, maternal mental illness, statement of SEN, and prior (i.e., at age 11) mental health difficulties (measured with the SDQ, as discussed). In the fourth step, model (4), we controlled additionally for whether the cohort member slept in a shared bedroom, the frequency of family meals, whether or not the cohort member had moved school, how long falling asleep took over the last 4 weeks, how frequently was sleep disturbed during the night over the last 4 weeks, as well as area safety and air pollution (NO_2_). In the fifth and final step, model (5), we added the interaction term, *b*_*int*_ = TTS × Sex.

Missing data were imputed using multiple imputation by chained equations (Raghunathan et al., 2001), and the imputed datasets were combined following Rubin's rules (Rubin, 1987). This method of imputing data from large surveys has been established as the most robust, especially for epidemiological studies (Azur et al., 2011). All calculations were performed using R (R Core Team, 2021) with the “mice” package (van Buuren & Groothuis‐Oudshoorn, 2011), R version 4.1.1 (2021‐08‐10); for reproducibility, we note that we performed *m* = 25 imputation runs with seed *s* = 347, and confirmed that the results remain unaffected by increasing imputation numbers (*m* = 50, 75, 100). The analysis was carried out separately for TTS on school nights, TTS on non‐school nights, and the inconsistency between them.

#### Sensitivity analysis

In the last stage of the analysis, we excluded adolescents who slept under 7 h or over 11 h on a school night—this led to the exclusion of 1685 cohort members (15% of the analytic sample). Adolescents sleeping between 7 and 11 h were within an hour of the recommended sleep duration range for their age group (Paruthi et al., 2016). For non‐school nights, we also calculated a sleep midpoint which we entered alongside sleep duration in our models instead of TTS (for complete cases only in a parsimonious model without excluding over‐ and under‐sleepers, since sleep duration was controlled for in this model).

## RESULTS

### Sample bias

The analytic sample included the vast majority (97%) of adolescents in the MCS sweep at age 14 and was balanced in terms of sex (50% female). The roughly 400 cohort members (3%) who were excluded from the analytic sample due to incomplete responses on the TTS variable were mostly males (61%), of lower incomes (Cohen's *d* = −0.43), from lower education backgrounds (*d* = −0.39), with lower word scores (*d* = −0.48) and poorer mental health at age 11 (*d* = 0.53). Table 1 includes some of the relevant details in terms of sample bias, and the SOM includes details for all variables (SOM, 2023).

#### Missing values

In the analytic sample, the area stratum, sex, and independent (TTS) variables had no missing values at all. Ethnicity had 93 (<1%) missing values, income only had 10 missing values, and several covariates had similarly low missingness of less than 1% (time it takes to fall asleep, trouble staying asleep, fruit and vegetable consumption, statemented SEN, screen time, and physical activity). Most other covariates had missing values in the range of 100–1000, namely, up to 9%. The only covariates to exceed this were: frequency of family meals, bedroom sharing status and chronic illness (1165 missing values each), perceived area safety (1174), and whether the cohort member had moved school (1201). The highest level of missingness occurred for maternal mental illness, with 2016 (18%) missing values. We established that missing data were MAR, as opposed to missing completely at random, by comparing systematic missingness in the observed data. That is, we performed bivariate analyses between the group with complete records, that is, without any missingness, and the group of records with at least one missing entry, the results of which are presented in full in the supplemental material (SOM, 2023). These analyses enabled us to proceed with the imputation process, and to use *m* = 25 runs in each case.

### Plot and correlations

**FIGURE 1:**
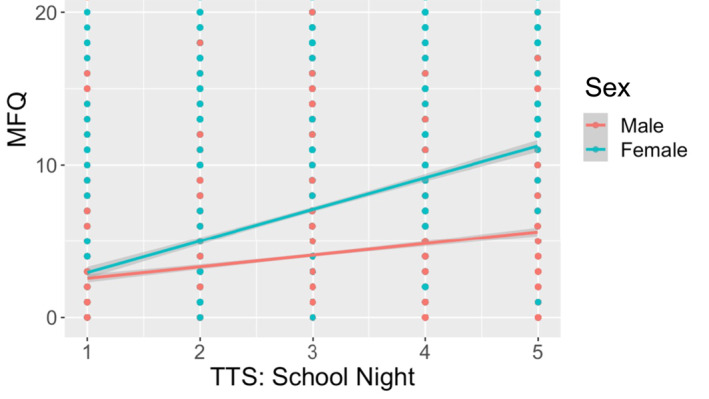
Mean score of depressive symptoms (MFQ) against time‐to‐sleep (TTS) on a school night, stratified by sex (unweighted, complete cases, *n* = 11,026). TTS, time‐to‐sleep.

As can be seen in the scatterplot of the mean score for depressive symptoms (MFQ) against self‐reported TTS on a school night (Figure 1), it appears that adolescents with “morning” chronotypes (TTS less than or equal to 2) had the lowest total mean MFQ scores, while adolescents with “evening” chronotypes (TTS more than or equal to 4) had the highest scores. This positive relationship seemed linear, and apparently moderated by sex with an “evening” chronotype more strongly associated with depressive symptoms in females. Similar results were also obtained for non‐school nights (SOM, 2023).

In terms of correlations among the covariates, the strongest positive correlation was observed between income and maternal education, and the strongest negative correlation was between income and prior mental health difficulties at age 11 years—see Table 2 for a summary of the key correlations, and the full table for all pairs in the SOM (2023). The strength of these correlations did not pose multicollinearity issues, something that we checked separately through a Variance Inflation Factor (VIF) analysis (all ratio and interval variables had VIF <5).

**TABLE 2:** Correlation coefficients matrix for the key study variables.

	(1)	(2)	(3)	(4)	(5)	(6)	(7)	(8)	(9)	(10)
Prior mental health problems (1)										
TTS: Non‐school night (2)	0.03									
Total MFQ (3)	**0.14**	**0.18**								
Night awakenings (4)	**0.15**	**0.07**	**0.38**							
TTS: School night (5)	**0.02**	**0.63**	**0.23**	**0.09**						
Screen time (6)	**0.05**	**0.19**	**0.13**	**0.06**	**0.14**					
Sleep latency (7)	**0.07**	**0.16**	**0.30**	**0.32**	**0.21**	**0.08**				
Peer victimization (8)	**0.15**	**0.01**	**0.39**	**0.17**	**0.05**	**0.04**	**0.16**			
Word score (9)	**−0.18**	−0.03	0.02	**−0.07**	**0.04**	**−0.05**	**0.06**	**0.06**		
Maternal education (10)	**−0.21**	**−0.09**	−0.01	**−0.08**	−0.01	**−0.07**	0.03	**0.05**	**0.25**	
Income (11)	**−0.27**	**−0.14**	−0.04	**−0.13**	**−0.07**	**−0.08**	−0.01	0.03	**0.25**	**0.57**

*Note*: Pearson's correlation coefficients (bold for ***p* < 0.05**).

### Regression models

For the main analysis, we first present the full results for models (1) to (4) for school nights. The results from the fully adjusted model (5), which includes the interaction term, *b*_*int*_ = TTS × Sex, are considered next for all three independent TTS variables (school night, non‐school night, and inconsistency). Finally, we present the sensitivity analysis for models (4) and (5) after excluding 1685 (15%) of the analytic sample who were either under‐sleeping or over‐sleeping.

*TTS on school nights*: In all four models, an "evening” chronotype on school nights was positively associated with depressive symptoms (total MFQ score), even after adjustment (as per model 4), with *b*_1_ = 0.748, *t*(11,276) = 10.098, *p* < 0.001, 95% CI [0.602, 0.893] (*cf*. Table 3). Model 4 explains 36% of the total MFQ score.

**TABLE 3:** Unstandardized regression coefficients (standard errors) for MFQ score in models (1) to (4) in relation to TTS: school night (imputed, weighted data).

	Model 1	Model 2	Model 3	Model 4
(Intercept)	2.013*** (0.251)	1.828*** (0.437)	−2.656** (0.933)	−3.752*** (1.018)
TTS				
School night	1.392*** (0.082)	1.331*** (0.080)	0.945*** (0.080)	0.748*** (0.074)
Stratum				
England—Disadvantaged	−0.230 (0.207)	−0.449* (0.197)	−0.172 (0.171)	−0.131 (0.164)
England—Ethnic	−1.055*** (0.207)	−0.651* (0.315)	0.033 (0.261)	0.016 (0.263)
Wales—Advantaged	−0.273 (0.347)	−0.282 (0.340)	−0.188 (0.287)	−0.296 (0.290)
Wales—Disadvantaged	−0.295 (0.228)	−0.693** (0.238)	−0.254 (0.218)	−0.294 (0.230)
Scotland—Advantaged	−1.026*** (0.293)	−0.988*** (0.266)	−0.846** (0.276)	−0.916** (0.288)
Scotland—Disadvantaged	−0.753* (0.338)	−1.003** (0.322)	−0.774** (0.298)	−0.864*** (0.257)
Northern Ireland—Advantaged	−1.319*** (0.364)	−1.322*** (0.327)	−0.330 (0.302)	−0.295 (0.281)
Northern Ireland—Disadvantaged	−1.581*** (0.338)	−2.043*** (0.331)	−1.192*** (0.284)	−0.943** (0.299)
Female		2.916*** (0.147)	3.021*** (0.130)	2.475*** (0.127)
Ethnicity				
Mixed		0.375 (0.372)	0.548 (0.319)	0.463 (0.304)
Indian		−1.228*** (0.334)	−0.239 (0.329)	−0.055 (0.325)
Pakistani and Bangladeshi		−1.749*** (0.334)	−0.514 (0.278)	−0.267 (0.270)
Black or Black British		−1.316*** (0.359)	−0.535 (0.332)	−0.403 (0.312)
Other Ethnic group (inc. Chinese)		−0.229 (0.578)	0.469 (0.493)	0.472 (0.460)
Income		−0.368*** (0.096)	−0.013 (0.088)	0.067 (0.084)
Maternal education		0.078 (0.141)	0.024 (0.117)	0.003 (0.108)
Word score			0.049 (0.036)	0.055 (0.033)
Quality of decision‐making			−0.007 (0.008)	−0.002 (0.007)
Risk‐taking			0.142*** (0.031)	0.089** (0.029)
Chronic illness			0.864*** (0.203)	0.574** (0.191)
BMI			0.001*** (0.000)	0.001*** (0.000)
Fruit intake			−0.423** (0.136)	−0.394** (0.122)
Vegetable intake			−0.312* (0.126)	−0.226 (0.123)
Physical activity			−0.139* (0.064)	−0.183** (0.059)
Screen time			0.145** (0.045)	0.140*** (0.041)
Alcohol consumption			0.966*** (0.105)	0.846*** (0.103)
Season				
Spring			−0.440* (0.203)	−0.428* (0.192)
Summer			−0.215 (0.230)	−0.194 (0.222)
Winter			−0.311 (0.223)	−0.419 (0.214)
Peer victimization			1.381*** (0.063)	1.184*** (0.059)
No maternal mental illness			−0.637*** (0.176)	−0.568*** (0.162)
Statemented SEN			0.286 (0.558)	0.118 (0.502)
Prior mental health problems (age 11)			0.078* (0.032)	0.056 (0.029)
Shared bedroom				−0.290 (0.167)
Family meals				−0.009 (0.088)
Moved school				0.355 (0.283)
Sleep latency				0.402*** (0.060)
Night awakenings				0.970*** (0.049)
Area safety				−0.126 (0.160)
Air pollution (NO_2_)				−0.051 (0.026)
*R* ^2^	0.057	0.120	0.297	0.363
Adjusted *R* ^2^	0.056	0.119	0.295	0.360
*N*_observations_	11,314	11,314	11,314	11,314

Abbreviations: MFQ, mood and feelings questionnaire; TTS, time‐to‐sleep.

***p < 0.001; **p < 0.01; *p < 0.05.

*TTS*: *non‐school nights*: Evening chronotype (TTS: non‐school night) was positively associated with the total MFQ score, even in the fully adjusted model (4), *b*_1_ = 0.631, *t*(11,276) = 7.762, *p* < 0.001, 95% CI [0.471, 0.790].

*TTS inconsistency*: Inconsistency in TTS was negatively associated with the total MFQ, even in the fully adjusted model (4), *b*_1_ = −0.199, *t*(11,276) = −2.368, *p* = 0.018, 95% CI [−0.364,−0.034].

*Sex and TTS interaction*: The fully adjusted model which also includes the term of the interaction between TTS and sex was fitted to test our hypothesis that the association between an evening chronotype and depressive symptoms would be larger in females. For the three cases of TTS on a school night, TTS on a non‐school night, and the inconsistency in TTS between them, we found a large and significant moderation effect and in the direction that we expected, as shown in Table 4.

**TABLE 4:** Unstandardized coefficients (standard errors) for MFQ model (5) by TTS: school night, non‐school night, and inconsistency (imputed, weighted data).

	School night	Non‐school night	Inconsistency
(Intercept)	−1.934 (1.034)	−2.263* (1.115)	−1.812 (1.093)
TTS	0.231* (0.090)	0.197* (0.091)	−0.037 (0.100)
Stratum			
England—Disadvantaged	−0.135 (0.160)	−0.175 (0.165)	−0.124 (0.164)
England—Ethnic	0.014 (0.263)	−0.014 (0.264)	−0.009 (0.263)
Wales—Advantaged	−0.299 (0.289)	−0.288 (0.290)	−0.259 (0.295)
Wales—Disadvantaged	−0.319 (0.233)	−0.299 (0.231)	−0.218 (0.228)
Scotland—Advantaged	−0.948** (0.296)	−0.906** (0.312)	−0.613* (0.298)
Scotland—Disadvantaged	−0.902*** (0.256)	−0.790** (0.261)	−0.541* (0.253)
Northern Ireland—Advantaged	−0.319 (0.274)	−0.342 (0.290)	0.010 (0.286)
Northern Ireland—Disadvantaged	−1.001*** (0.296)	−0.956** (0.312)	−0.584 (0.300)
Female	−0.793* (0.394)	−1.246* (0.538)	2.816*** (0.205)
Ethnicity			
Mixed	0.469 (0.306)	0.446 (0.300)	0.480 (0.307)
Indian	−0.031 (0.328)	−0.053 (0.326)	−0.062 (0.345)
Pakistani and Bangladeshi	−0.319 (0.267)	−0.315 (0.279)	−0.276 (0.261)
Black or Black British	−0.417 (0.300)	−0.533 (0.307)	−0.501 (0.307)
Other Ethnic group (inc. Chinese)	0.452 (0.460)	0.511 (0.481)	0.557 (0.467)
Income	0.053 (0.083)	0.054 (0.083)	0.011 (0.083)
Maternal education	0.007 (0.107)	0.031 (0.109)	0.012 (0.109)
Word score	0.053 (0.033)	0.074* (0.035)	0.069 (0.036)
Quality of decision‐making	−0.002 (0.007)	−0.002 (0.008)	−0.003 (0.007)
Risk‐taking	0.086** (0.029)	0.090** (0.029)	0.123*** (0.029)
Chronic illness	0.608** (0.191)	0.667*** (0.191)	0.607** (0.193)
BMI	0.001*** (0.000)	0.001** (0.000)	0.001*** (0.000)
Fruit intake	−0.381** (0.120)	−0.412*** (0.121)	−0.516*** (0.120)
Vegetable intake	−0.238 (0.122)	−0.257* (0.122)	−0.327** (0.123)
Physical activity	−0.179** (0.058)	−0.169** (0.059)	−0.186** (0.059)
Screen time	0.133*** (0.040)	0.134*** (0.040)	0.191*** (0.041)
Alcohol consumption	0.809*** (0.103)	0.830*** (0.104)	0.991*** (0.104)
Season			
Spring	−0.439* (0.191)	−0.429* (0.190)	−0.472* (0.192)
Summer	−0.211 (0.221)	−0.186 (0.220)	−0.181 (0.224)
Winter	−0.434* (0.209)	−0.414 (0.211)	−0.431* (0.216)
Peer victimization	1.168*** (0.059)	1.168*** (0.060)	1.173*** (0.060)
No maternal mental illness	−0.564*** (0.158)	−0.575*** (0.163)	−0.593*** (0.166)
Statemented SEN	0.005 (0.496)	0.029 (0.500)	−0.122 (0.507)
Prior mental health problems (age 11)	0.058 (0.029)	0.058 (0.031)	0.054 (0.031)
Shared bedroom	−0.321 (0.166)	−0.325 (0.167)	−0.396* (0.167)
Family meals	−0.014 (0.087)	−0.016 (0.085)	−0.050 (0.085)
Moved school	0.372 (0.284)	0.363 (0.284)	0.393 (0.285)
Sleep latency	0.388*** (0.059)	0.434*** (0.061)	0.492*** (0.059)
Night awakenings	0.960*** (0.049)	0.960*** (0.049)	0.976*** (0.049)
Area safety	−0.132 (0.160)	−0.146 (0.173)	−0.120 (0.184)
Air pollution (NO_2_)	−0.051* (0.026)	−0.060* (0.026)	−0.042 (0.027)
TTS × female	1.121*** (0.133)	0.950*** (0.136)	−0.365* (0.150)
*R* ^2^	0.368	0.361	0.350
Adjusted *R* ^2^	0.366	0.359	0.347
*N*_observations_	11,314	11,314	11,314

Abbreviations: MFQ, mood and feelings questionnaire; TTS, time‐to‐sleep.

***p < 0.001; **p < 0.01; *p < 0.05.

#### Sensitivity analysis

##### Excluding under‐sleepers and over‐sleepers

We carried out a sensitivity analysis to disassociate chronotype from sleep duration. Excluding adolescents who slept less than 7 h (or over 11 h) on a school night did not change the main effect of TTS. In the fully adjusted models (either with or without the TTS × sex interaction) there was a large and significant main effect of TTS and a moderation effect by sex as well. However, both the main and the moderation effects in the case of TTS inconsistency were found to be sensitive to the exclusion of under‐sleepers and over‐sleepers. Table 5 includes the results for the main effects in all three cases after full adjustment but without the TTS × sex interaction (model 4).

**TABLE 5:** Sensitivity analysis (excluding under‐sleepers and over‐sleepers); unstandardized coefficients (standard errors) for MFQ model (4) by TTS: school night, non‐school night, and inconsistency (imputed, weighted data).

	School night	Non‐school night	Inconsistency
(Intercept)	−2.871** (1.021)	−3.267** (1.080)	−1.412 (1.046)
TTS	0.562*** (0.091)	0.502*** (0.087)	0.059 (0.093)
Stratum			
England—Disadvantaged	−0.017 (0.177)	−0.048 (0.178)	−0.022 (0.177)
England—Ethnic	0.081 (0.288)	0.050 (0.288)	0.040 (0.291)
Wales—Advantaged	−0.159 (0.310)	−0.170 (0.313)	−0.099 (0.318)
Wales—Disadvantaged	−0.153 (0.242)	−0.141 (0.242)	−0.075 (0.243)
Scotland—Advantaged	−0.716* (0.292)	−0.712* (0.294)	−0.496 (0.297)
Scotland—Disadvantaged	−0.653* (0.285)	−0.648* (0.291)	−0.431 (0.290)
Northern Ireland—Advantaged	−0.109 (0.325)	−0.145 (0.338)	0.103 (0.336)
Northern Ireland—Disadvantaged	−0.642* (0.316)	−0.631* (0.321)	−0.364 (0.322)
Female	2.191*** (0.127)	2.208*** (0.128)	2.166*** (0.128)
Ethnicity			
Mixed	0.656 (0.368)	0.611 (0.365)	0.644 (0.368)
Indian	0.189 (0.388)	0.137 (0.379)	0.166 (0.395)
Pakistani and Bangladeshi	−0.120 (0.286)	−0.132 (0.293)	−0.077 (0.286)
Black or Black British	−0.386 (0.341)	−0.504 (0.331)	−0.480 (0.334)
Other ethnic group (inc. Chinese)	0.344 (0.460)	0.369 (0.464)	0.421 (0.460)
Income	0.067 (0.104)	0.075 (0.105)	0.042 (0.104)
Maternal education	0.012 (0.161)	0.027 (0.166)	0.022 (0.165)
Word score	0.032 (0.047)	0.041 (0.049)	0.042 (0.049)
Quality of decision‐making	−0.002 (0.007)	−0.002 (0.007)	−0.003 (0.007)
Risk‐taking	0.087** (0.032)	0.082* (0.032)	0.101** (0.031)
Chronic illness	0.653*** (0.190)	0.698*** (0.192)	0.678***(0.192)
BMI	0.001* (0.000)	0.000 (0.000)	0.001* (0.000)
Fruit intake	−0.397** (0.138)	−0.387** (0.139)	−0.451** (0.139)
Vegetable intake	−0.229 (0.118)	−0.238* (0.117)	−0.303** (0.117)
Physical activity	−0.220*** (0.060)	−0.219*** (0.060)	−0.223*** (0.061)
Screen time	0.118* (0.046)	0.106* (0.045)	0.139** (0.045)
Alcohol consumption	0.805*** (0.119)	0.778*** (0.121)	0.899*** (0.121)
Season			
Spring	−0.348 (0.194)	−0.347 (0.190)	−0.372 (0.190)
Summer	−0.088 (0.228)	−0.078 (0.225)	−0.078 (0.226)
Winter	−0.465* (0.207)	−0.462* (0.205)	−0.479* (0.206)
Peer victimization	1.134*** (0.068)	1.136*** (0.068)	1.121*** (0.069)
No maternal mental illness	−0.482** (0.160)	−0.471** (0.162)	−0.486** (0.164)
Statemented SEN	0.194 (0.545)	0.177 (0.543)	0.058 (0.544)
Prior mental health problems (age 11)	0.060*** (0.016)	0.060*** (0.016)	0.057*** (0.016)
Shared bedroom	−0.308 (0.181)	−0.308 (0.180)	−0.376* (0.180)
Family meals	0.033 (0.107)	0.037 (0.108)	0.009 (0.112)
Moved school	0.395 (0.291)	0.421 (0.289)	0.438 (0.292)
Sleep latency	0.393*** (0.067)	0.404*** (0.068)	0.438*** (0.066)
Night awakenings	0.954*** (0.053)	0.947*** (0.052)	0.948*** (0.053)
Area safety	−0.131 (0.176)	−0.130 (0.181)	−0.132 (0.190)
Air pollution (NO_2_)	−0.044 (0.028)	−0.050 (0.028)	−0.041 (0.028)
*R* ^2^	0.326	0.324	0.319
Adjusted *R* ^2^	0.323	0.321	0.316
*N*_observations_	9629	9629	9629

Abbreviations: MFQ, mood and feelings questionnaire; TTS, time‐to‐sleep.

***p < 0.001; **p < 0.01; *p < 0.05.

Table 6 includes the results for the main and moderation effects only (model 5). Full details for all models are included in the SOM (SOM, 2023). As seen in Table 6, in contrast to the school night and non‐school night TTS variables, for TTS inconsistency there was no moderation by sex.

**TABLE 6:** Sensitivity analysis (excluding under‐sleepers and over‐sleepers); unstandardized coefficients (standard errors) for the main and interaction term effects in MFQ model (5) by each of the three cases of TTS (imputed, weighted data).

	School night	Non‐school night	Inconsistency
TTS	0.144 (0.106)	0.153 (0.095)	0.026 (0.102)
Female	−0.325 (0.446)	−0.740 (0.575)	2.083*** (0.224)
TTS × female	0.919*** (0.155)	0.766*** (0.145)	0.075 (0.170)
*N*_observations_	9629	9629	9629

Abbreviations: MFQ, mood and feelings questionnaire; TTS, time‐to‐sleep.

***p < 0.001; **p < 0.01; *p < 0.05.

##### Modeling results by sleep midpoint and sleep duration

Table 7 presents the results of the second sensitivity analysis, for the complete cases. This used, for non‐school nights, the sleep midpoint and sleep duration (instead of TTS), while also adjusting for confounders (and since we controlled for sleep duration, the exclusion of under‐ and over‐sleepers was not necessary in this case). As can be seen, even after adjustment for sleep duration, a later sleep midpoint was associated with more depressive symptoms. Full numerical accuracy is retained in this table, for illustration, as also appears in the supplemental material (SOM, 2023).

**TABLE 7:** Sensitivity analysis: MFQ by the midpoint of sleep for non‐school nights, adjusted for sleep duration (survey‐weighted, fully adjusted, complete *N* = 6579).

	Estimate	Std. error	*t* value	Pr(>|*t*|)
(Intercept)	−4.7382599	1.0366617	−4.5706907	0.0000068
Sleep midpoint				
Non‐school night	0.4884522	0.1039753	4.6977694	0.0000038
Sleep duration	−0.3436188	0.0813625	−4.2233060	0.0000308
Stratum				
England—Disadvantaged	−0.0646575	0.2115902	−0.3055788	0.7601086
England—Ethnic	0.1765235	0.3630937	0.4861652	0.6271569
Wales—Advantaged	0.1289814	0.3469743	0.3717319	0.7103194
Wales—Disadvantaged	−0.0765655	0.2414566	−0.3170982	0.7513598
Scotland—Advantaged	−0.8520811	0.3561352	−2.3925773	0.0172618
Scotland—Disadvantaged	−0.6067659	0.3495789	−1.7357051	0.0835037
Northern Ireland—Advantaged	−0.1061248	0.3631757	−0.2922135	0.7702980
Northern Ireland—Disadvantaged	−0.6925008	0.3564818	−1.9425979	0.0528734
Female	2.4625849	0.1395488	17.6467674	0.0000000
Ethnicity				
Mixed	0.4468715	0.4182687	1.0683837	0.2860901
Indian	−0.6448487	0.4439521	−1.4525188	0.1472610
Pakistani and Bangladeshi	−0.4822046	0.3744122	−1.2878977	0.1986398
Black or Black British	0.1360923	0.5316831	0.2559651	0.7981294
Other ethnic group (inc. Chinese)	0.1309435	0.5864825	0.2232693	0.8234573
Income	0.0276327	0.0833309	0.3316020	0.7403900
Maternal education	0.0014824	0.0657710	0.0225393	0.9820307
Word score	0.1048507	0.0273335	3.8359810	0.0001485
Quality of decision‐making	0.0053944	0.0064602	0.8350190	0.4042816
Risk‐taking	0.1293808	0.0372644	3.4719717	0.0005819
Chronic illness	0.6238654	0.2281415	2.7345545	0.0065671
BMI	0.0004890	0.0002009	2.4345424	0.0154142
Fruit intake	−0.5212793	0.1451667	−3.5909003	0.0003771
Vegetable intake	−0.2663625	0.1474437	−1.8065369	0.0717007
Physical activity	−0.2298917	0.0707441	−3.2496231	0.0012686
Screen time	0.1491790	0.0477924	3.1213943	0.0019511
Alcohol consumption	0.7267669	0.1253933	5.7958974	0.0000000
Season				
Spring	−0.3727448	0.2686129	−1.3876649	0.1661295
Summer	−0.1627347	0.2937030	−0.5540792	0.5798815
Winter	−0.7890732	0.3096546	−2.5482366	0.0112572
Peer victimization	1.3097305	0.0633445	20.6763138	0.0000000
No maternal mental illness	−0.5198229	0.1840146	−2.8248996	0.0050030
Statemented SEN	−0.0894127	0.4324977	−0.2067356	0.8363376
Prior mental health problems (age 11)	0.0615274	0.0153663	4.0040414	0.0000762
Shared bedroom	−0.4325645	0.2197108	−1.9687904	0.0497727
Family meals	−0.0504460	0.0852023	−0.5920732	0.5541871
Moved school	0.3725152	0.3534986	1.0537956	0.2927097
Sleep latency	0.5220423	0.0735463	7.0981406	0.0000000
Night awakenings	1.1493966	0.0675958	17.0039652	0.0000000
Area safety	−0.0412725	0.1328766	−0.3106076	0.7562855
Air pollution (NO_2_)	−0.0153020	0.0337934	−0.4528094	0.6509690

Abbreviation: MFQ, mood and feelings questionnaire.

It should be noted that the sex × chronotype interaction remains statistically significant even in this case, namely, when the independent variable is the midpoint of sleep on non‐school nights and we adjust for sleep duration and all the other confounders, *b*_femalexmidpoint_ = 0.851, *t*(6578) = 4.117, *p* < 0.001. Finally, we highlight that the TTS measure (TTS: non‐school night) and the equivalent sleep midpoint measure (for non‐school nights) are strongly correlated, with *r* = 0.79, *t*(10,430) = 133, *p* < 0.001 (the correlation is even higher for school nights). Therefore, TTS and sleep midpoint on either school nights or non‐school nights are both valid proxies to chronotype in this age group.

## DISCUSSION

The present study tested the association between chronotype and depressive symptoms at age 14 years in a large, general‐population sample in the UK after adjustment for a wide range of potential confounders. Chronotype was approximated by TTS, namely, the adolescents' time range in which they actually fell asleep.^1^ The study also explored if the association differs by sex, and in a supplementary analysis it disassociated chronotype from sleep duration. In all cases, the exposures were TTS on a school night, TTS on a non‐school night, and the TTS inconsistency between school nights and non‐school nights.

The first finding was a significant association between eveningness and depressive symptoms even after full adjustment for covariates at the level of the individual, family, and neighborhood, and even after excluding adolescents who were either under‐sleeping or over‐sleeping. The second finding was that sex moderates the evening chronotype‐depressive symptoms association, with males being more protected against the impact of eveningness. A main effect and a moderation effect were also found for TTS inconsistency, where a greater TTS difference (Δ ≥ 1) between non‐school nights and school nights was negatively associated with depressive symptoms. This finding can be interpreted in terms of the expected shift to eveningness in this age group: a positive Δ is in line, developmentally, with this shift to a later chronotype, whereas a negative Δ is not. In this sense, it could be argued that adolescent sleep schedules that change between school and non‐school nights into a more evening‐type chronotype (i.e., when “allowed”) are associated with fewer depressive symptoms. However, it should be noted that when under‐sleeping or over‐sleeping adolescents were excluded from the analysis, the strength of both effects (direct and moderating) reduced in size and became non‐significant, which implies that this finding is mostly relevant to those in the extremes of the sleep duration distribution.

The fact that the association between TTS inconsistency and depressive symptoms was non‐significant in the sensitivity analysis (i.e., when under‐ and over‐sleepers were excluded) implies that extreme values on this TTS variable track suboptimal sleep duration, related to sleep debt (Shen et al., 2021), which is “repaid” during non‐school mornings (Misiunaite et al., 2020), or that adolescents are more aligned with their natural circadian rhythm at the weekend. In contrast to current sleep recommendations (Moore & Meltzer, 2008), which suggest that TTS inconsistency be minimized, the evidence presented here suggests that this is not the case, and that greater inconsistency is associated with fewer depressive symptoms for adolescents who are sleep deprived. In turn, this may suggest that risk of depression in adolescence is reduced when individual sleep patterns align with the normative shift towards eveningness during this age period. Interestingly, we found that prior mental health difficulties (at age 11) had a significant association with depressive symptoms at age 14 only in the sensitivity analysis, that is, only when we retained adolescents whose sleep duration was within the recommended range (±1 h). This points to the potential mediating role of sleep duration in the association between prior mental health difficulties and future depression.

However, the findings of the present study are constrained in view of several limitations. First, even though we considered prior mental health difficulties as a potential confounder, the results of the study remain correlational in nature. Second, both the independent and dependent variables were self‐reported measures, namely, there were neither actigraphy measurements (for sleep) nor clinical assessments (for depressive symptoms). In addition, there was no clinical screening for sleep disorders available. Third, the study was limited by TTS ranges that were 1 h long, or longer (for instance, “before 9 p.m,” or “after midnight”). Fourth, the wider TTS ranges (such as, “after midnight”) pose another problem in terms of imprecise measurement—there is arguably a substantial difference between typically going to sleep around 12:30 a.m, for example, versus around 2 a.m. Fifth, the previous two limitations also imply that our TTS inconsistency measure is also imprecise as it is calculated using the difference in TTS between weekdays and weekends. As a result, the preference for much later sleep times cannot be determined from our data, given such measurement imprecision. However, we must note that we made full use of the data available to approximate chronotype as well as possible. For example, in a sensitivity analysis, we calculated a potentially more robust proxy measure of chronotype, the midpoint between TTS and time of waking up, for non‐school nights. This sensitivity analysis also fully confirmed our findings. That is, an evening chronotype, even after accounting for sleep duration, was positively related to depressive symptoms in adolescence.

In conclusion, and despite some important limitations, this study presented evidence on the association between eveningness and depressive symptoms in adolescence in the general population. The association was found to be stronger in females and was robust even after controlling for a wide range of environmental and biopsychosocial factors, both proximal and distal. Future research should address the limitations we highlighted and test the patterns we identified across cultures and developmental windows, during the full adolescence period.

## AUTHOR CONTRIBUTIONS

**Dimitris I. Tsomokos**: Conceptualization, formal analysis, investigation, methodology, software, visualization, writing – original draft, writing – review & editing. **Elizabeth Halstead**: Conceptualization, methodology, writing – review & editing. **Eirini Flouri**: Conceptualization, formal analysis, funding acquisition, methodology, project administration, supervision, writing – review & editing.

## CONFLICT OF INTEREST STATEMENT

The authors have declared that they have no competing or potential conflicts of interest.

## ETHICAL CONSIDERATIONS

This study uses secondary data analysis of the Millennium Cohort Study (MCS) data. The MCS data is publicly available from the UK Data Service, under license, and its use does not require additional ethics approval; the primary data collection and public availability of data, for each survey wave, was subject to Multi‐Centre Ethics Committees led by the National Health Service in the United Kingdom. For more information on the MCS ethics approvals and related processes, consult for example, the UCL Institute of Education “Ethical review and consent” (2012) document, available online: https://cls.ucl.ac.uk/wp‐content/uploads/2017/07/MCS‐Ethical‐review‐and‐consent‐Shepherd‐P‐November‐2012.pdf.

## Data Availability

The data that support the findings of this study are openly available in UK Data Service at http://doi.org/10.5255/UKDA‐Series‐2000031, reference number SN: 2000031.

## ENDNOTE

^1^
We stress that this time‐to‐sleep (TTS) variable is the “clock time” of when a cohort member started their sleep, not the time it took them to fall asleep (which is a different variable that we have controlled for, that is, sleep latency).
